# Hyperspectral imaging and dynamic selective peak transformer for early-stage classification of lettuce heat responses

**DOI:** 10.3389/fpls.2026.1890104

**Published:** 2026-07-14

**Authors:** Minglu Tian, Hui Yang, Mengen Yuan, Songtao Ban, Dong Hu, Jifeng Ning, Linyi Li, Guotao Huo, Jiahui Qi, Shuping He, Guojun Ge, Shiwei Wei, Tian Xia

**Affiliations:** 1 Institute of Agricultural Science and Technology Information, Shanghai Academy of Agricultural Sciences, Shanghai, China; 2 Key Laboratory of Intelligent Agricultural Technology (Yangtze River Delta), Ministry of Agriculture and Rural Affairs, Shanghai, China; 3 School of Computer and Information Engineering, Shanghai Polytechnic University, Shanghai, China; 4 College of Information Engineering, Northwest A&F University, Yangling, Shaanxi, China; 5 Shaanxi Engineering Research Center of Agricultural Information Intelligent Perception and Analysis, Yangling, Shaanxi, China; 6 Chinese Academy of Sciences (CAS) Center for Excellence in Molecular Plant Sciences, Shanghai Institute of Plant Physiology and Ecology, Chinese Academy of Sciences, Shanghai, China; 7 Shanghai Agrobiological Gene Center, Shanghai, China

**Keywords:** deep learning, heat stress, high-throughput phenotyping, hyperspectral imaging, lettuce

## Abstract

**Introduction:**

Early-stage detection and classification of lettuce heat responses are essential for non-destructive phenotyping, yet conventional assessment mainly relies on visible symptoms and manual observation.

**Methods:**

This study constructed a lettuce hyperspectral dataset comprising heat-sensitive and heat-tolerant varieties under control and high-temperature treatments, and proposed the Dynamic Selective Peak Transformer (DSPformer). DSPformer integrates edge-enhanced feature extraction, dynamic multi-scale spatial-spectral representation, Peak-k selective attention, and a confusion-aware dynamic focal loss to enhance discriminative features while reducing spectral redundancy, class imbalance, and inter-class confusion.

**Results:**

Under the patch-level evaluation protocol, DSPformer achieved 96.22% accuracy, 95.55% recall, 96.35% precision, and 95.95% F1-score, outperforming the compared CNN- and Transformer-based models. Day-wise evaluation showed that DSPformer reached 82.61% accuracy on Day 1 and 96.55% on Day 3, before visible heat-stress symptoms appeared on Day 6. Under a plant-level partition protocol, DSPformer maintained robust performance with 93.76 +/- 0.49% accuracy. Additional evaluation on the Indian Pines benchmark further demonstrated the applicability of DSPformer to general hyperspectral image classification.

**Discussion:**

These findings suggest that hyperspectral imaging can capture heat-stress-sensitive information beyond visual phenotypes, and that DSPformer provides a promising framework for early, non-destructive lettuce heat-response screening and hyperspectral phenotyping-assisted breeding.

## 1 Introduction

Lettuce is one of the most widely cultivated leafy vegetables worldwide and has attracted increasing attention because of its high nutritional value, short growth cycle, and economic importance (Qin et al., 2025). Lettuce is highly sensitive to heat stress (Wei et al., 2024). Extended exposure to heat results in leaf tip burn, scorching, and quality deterioration, which severely limits its market quality and commercial value (Pereira et al., 2024). Screening and breeding heat-tolerant lettuce varieties is one of the most effective approaches to addressing the challenges posed by extreme climate conditions (Sandoya et al., 2024). Traditional screening of heat-tolerant germplasm relies on manual evaluation of heat-related phenotypes under high-temperature conditions, followed by metabolomic and phenomic analyses (Chen et al., 2022; Belisle et al., 2024). This process is time-consuming and costly, which limits its applicability in modern high-throughput breeding.

The advancement of high-throughput plant phenotyping (HTPP) technology has greatly enhanced the ability to evaluate plant traits efficiently and non-destructively (Ban et al., 2025). Hyperspectral imaging (HSI), as a key technology in HTPP analysis, enables non-contact acquisition of hundreds to thousands of spectral bands covering the visible (VIS) and near-infrared (NIR) regions, along with the reflectance spectrum of each pixel. By analyzing reflectance at different wavelengths, HSI combined with classical machine learning algorithms, including support vector machines (SVM) and random forests (RF) (Khan et al., 2022; Ryu et al., 2023), has been used to detect plant nutritional status (Pandey et al., 2023), heat stress (Faqeerzada et al., 2022; Zhang et al., 2025b), and disease stress (Zhang et al., 2024). However, these approaches rely on manually designed features, which may adversely affect model classification performance (Kumar et al., 2024).

Recent studies have increasingly combined deep learning with hyperspectral imaging for plant phenotyping, quality assessment, and stress detection (Sharma and Srivastava, 2025). Among them, convolutional neural networks (CNNs) have demonstrated strong capability in learning spectral-spatial representations from hyperspectral data and have achieved promising results in various agricultural applications (Bera et al., 2022; Yang et al., 2024; Phanomsophon et al., 2024; Cui et al., 2022). However, the locality of convolution operations limits their ability to model long-range spatial-spectral dependencies, which are important for hyperspectral image classification.

Transformer architectures alleviate some limitations of CNNs by modeling long-range dependencies through self-attention mechanisms (Vaswani et al., 2017). Since the introduction of Vision Transformer (ViT), Transformer-based models have achieved remarkable performance in hyperspectral image classification (Dosovitskiy et al., 2020). However, two challenges remain. First, subtle local spatial details may be weakened during global attention aggregation, which is particularly problematic for early-stage stress detection where phenotypic differences are extremely limited. Second, hyperspectral images contain substantial spectral redundancy, causing attention mechanisms to aggregate irrelevant responses and reducing feature discrimination (Ye et al., 2024). Therefore, developing an effective Transformer framework that can simultaneously preserve local spatial details and suppress redundant spectral information remains an important challenge for early stress classification.

In addition to model architecture, the design of the loss function also plays a critical role in determining classification performance (Zhang et al., 2025a). Conventional loss functions, such as cross-entropy (CE), often perform inadequately under class imbalance and noisy-label conditions (Henning et al., 2023). To address this problem, focal loss introduces a weighting factor τ and a modulating factor ${\left(1-p_{y}\right)}^{\gamma }$, thereby reducing the contribution of easily classified samples and increasing the focus on hard examples (Lin et al., 2018). Furthermore, calibration-aware adaptive focal loss (AdaFocal) dynamically adjusts γ to improve model calibration and alleviate the low-confidence issue associated with focal loss (Ghosh et al., 2022).

In summary, research integrating HSI with modern deep learning approaches in various agricultural fields has advanced beyond traditional machine learning techniques. However, existing methods mainly emphasize disease detection, nutritional assessment, or visible stress symptoms, few studies have investigated the use of deep learning for early recognition of heat tolerance in lettuce. Moreover, the deep learning algorithms and loss functions mentioned above still have certain limitations. CNNs tend to weaken detailed texture features (Bae et al., 2023), and their fixed receptive fields limit the extraction of multi-scale features (Jiao et al., 2023). The top-k mechanism overlooks the contribution of extreme negative values (Lv et al., 2024). Focal Loss and AdaFocal do not fully leverage the sample imbalance and class confusion characteristics inherent in each category.

This study proposes a hyperspectral imaging-based deep learning framework for early-stage classification of lettuce heat responses under controlled experimental conditions. The main contributions are summarized as follows:

1. An early-stage hyperspectral phenotyping framework is established for lettuce heat-response classification. By constructing a lettuce hyperspectral dataset under control and high-temperature treatments, this study investigates whether subtle spatial-spectral cues associated with early heat responses can be captured before visible symptoms become pronounced, providing a methodological basis for non-destructive early-stage heat-response screening.
2. DSPformer is proposed as a dynamic selective spatial-spectral representation network tailored for early heat-response classification. The model integrates edge-enhanced feature extraction, multi-scale dynamic convolution, dynamic activation, and Peak-k attention to strengthen subtle edge-texture and spectral-spatial representations while suppressing redundant hyperspectral information.
3. A confusion-aware dynamic focal loss is introduced and the proposed framework is comprehensively evaluated. The loss function adaptively balances class imbalance and inter-class similarity during training, while experiments on the self-constructed lettuce dataset and the public Indian Pines benchmark demonstrate the effectiveness and methodological applicability of DSPformer for hyperspectral image classification tasks.

## 2 Materials and methods

### 2.1 Experimental scheme

Lettuce varieties were selected from the Gene Resource Center of Shanghai Agricultural Biotechnology (SAGC), including two heat-sensitive varieties (A13 and W1063) and two heat-tolerant varieties (W1105 and K410), with 96 plants per variety used as experimental samples. Two treatments—high-temperature treatment (HT-Treatment) and control check (CK-Treatment)—were applied to each variety, with 48 plants assigned to each treatment. Uniform, healthy seeds were sown in germination trays lined with filter paper. After adding sufficient water to moisten the seeds, trays were incubated in a 20 °C constant-temperature chamber for 48 hours.

The growth substrate was prepared in a 4:1:1 volumetric ratio of peat, vermiculite, and perlite. Germinated seeds were sown in 128-cell trays, covered with vermiculite, watered appropriately, and then placed in a controlled-environment chamber. The chamber conditions were set as follows: a 14/10 h light/dark photoperiod, 23 °C/17 °C day/night temperatures, 70% relative humidity, and 350 ± 10 μmol·m−2·s−1 light intensity. When young plants reached the two-leaf stage, uniform seedlings were selected and transplanted into 6 cm × 6 cm pots.

After transplanting, seedlings assigned to the HT-Treatment were transferred to a controlled-environment chamber with elevated temperature conditions (day/night: 37 °C/32 °C), while CK-Treatment were maintained under normal temperature conditions (25 °C/20 °C). During the heat-treatment period, all plants were supplied with water under a normal irrigation regime to maintain adequate substrate moisture, and no drought treatment was imposed. The high-temperature treatment started at the two-leaf stage and lasted for 12 days (visible phenotypes appeared on day 6, specifically showing that, under HT-Treatment, the leaves of heat-sensitive varieties were smaller and paler than those under CK-Treatment, with leaf curling and damage). In this study, day 0 denotes the initial imaging time point at the onset of temperature treatment. All other environmental factors, including photoperiod, humidity, and light intensity, were kept identical between the two treatments.

### 2.2 Image collection and processing

**Figure 1:**
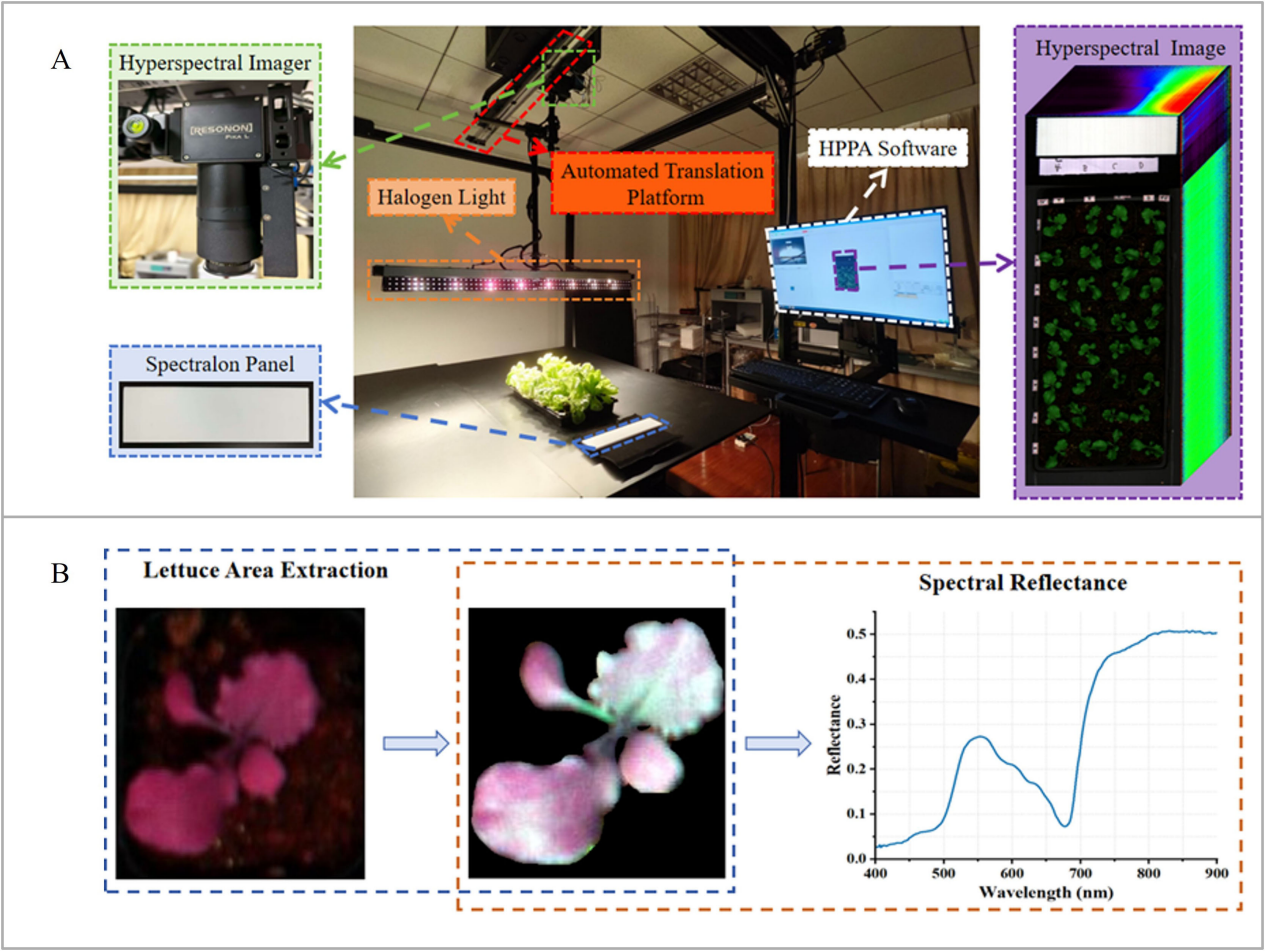
Hyperspectral images collection and processing. **(A)** Hyperspectral Imaging System and **(B)** processing process of lettuce hyperspectral image.

All lettuce hyperspectral images were captured in a dark environment in this study. During lettuce cultivation, hyperspectral images were obtained using a hyperspectral phenotyping system (**Figure 1A**) at days 0, 1, 2, 3, 4, 5, 6, 9, and 12. The system integrates an XY two-dimensional linear scanning stage, a linear uniform halogen lighting system, and a Pika L hyperspectral imager (Resonon Inc., Bozeman, Montana, USA). The imager spans a spectral range from 400 to 1000 nm at a 2.1 nm spectral resolution, comprising 300 spectral bands and 900 spatial channels. During imaging, a Spectralon reflectance panel was positioned adjacent to the lettuce specimens to serve as a white reference for each individual spectral scan.

SpectrononPro software (v3.1.1, Resonon Inc., Bozeman, Montana, USA) was used to correct the collected hyperspectral images and convert them to reflectance data, with ENVI software (v5.6, NV5 Geospatial, Broomfield, Colorado, USA) employed for separating lettuce plants from background regions. Python code was then developed to remove low signal-noise spectral data and extract mean reflectance spectra from the valid plant pixels. For each sample, the average reflectance was calculated across all valid plant pixels over the spectral range of 400–1000 nm. To provide a spectral interpretation of early heat responses, the extracted spectra were grouped according to treatment and heat-response category to compare the reflectance characteristics of CK- and HT-treated plants. **Figure 1B** illustrates the processing workflow.

### 2.3 Dataset construction

**Figure 2:**
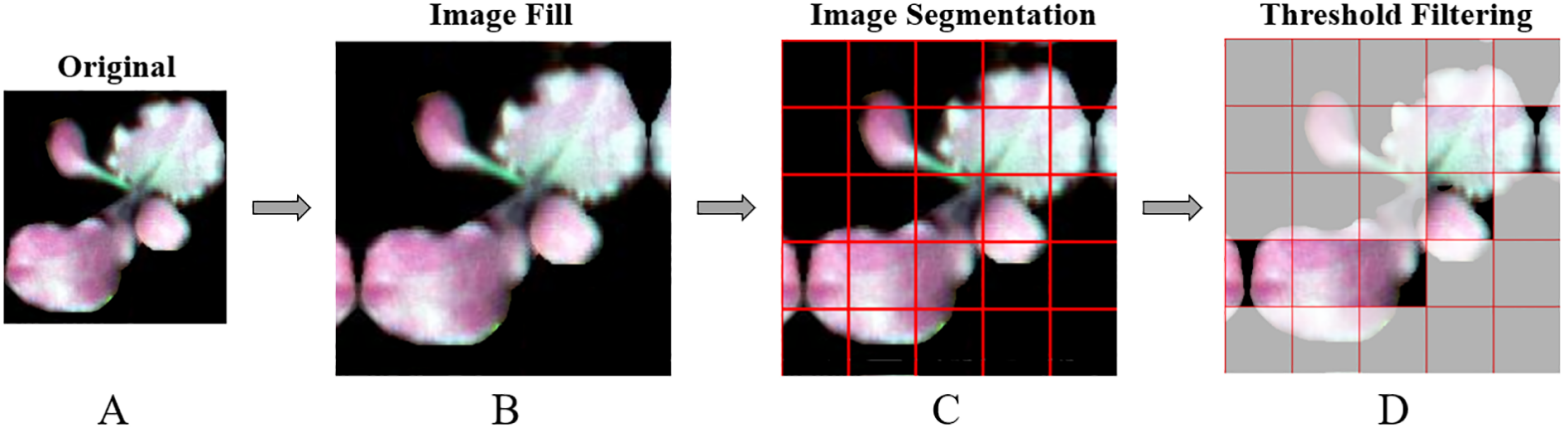
The process of cutting the original image into image blocks. **(A)** Acquired hyperspectral image; **(B)** image after symmetrical padding; **(C)** segmented image blocks; **(D)** image blocks after threshold filtering.

All lettuce samples were classified into four categories: heat-sensitive varieties under CK-Treatment (CK-Treatment-Sensitive, CTS), heat-sensitive varieties under HT-Treatment (HT-Treatment-Sensitive, HTS), heat-tolerant varieties under CK-Treatment (CK-Treatment-Tolerant, CTT), and heat-tolerant varieties under HT-Treatment (HT-Treatment-Tolerant, HTT). Lettuce hyperspectral images varied in size due to different varieties and multiple acquisition time points. Since most models require uniform input dimensions, the images were cropped into 64×64 pixels patches. Additionally, a pixel coverage threshold of 80% was applied to remove patches with insufficient lettuce coverage. **Figure 2** illustrates the process.

Before image segmentation, each image was symmetrically padded along height and width to the nearest multiple of 64, as shown in **Figure 2B**. This strategy facilitates image cropping and, compared to zero-padding, effectively reduces edge effects while better preserving leaf shape continuity and spatial and spectral information. **Figure 2C** presents the outcomes of dividing the padded images into 64×64 pixel patches. The transparent areas in **Figure 2D** indicate patches with less than 80% valid pixels, which were discarded during dataset construction.

After cropping and filtering, 2646 hyperspectral patches were obtained. Following common practice in hyperspectral image classification studies, a patch-level partition protocol was adopted for the primary benchmark experiments. The patches were randomly divided into training, validation, and test subsets at a ratio of 7:1:2. Data augmentation was applied only to the training subset using five geometric operations, including rotations of 90°, 180°, and 270°, and horizontal and vertical flipping. The same partition protocol was adopted for all compared models to ensure a consistent and fair evaluation setting. To further investigate the potential influence of correlations among patches originating from the same lettuce plant, an additional plant-level partition experiment was conducted, in which all patches extracted from a single plant were assigned exclusively to one subset.

### 2.4 Lettuce phenotypic observation in hyperspectral images

**Figure 3:**
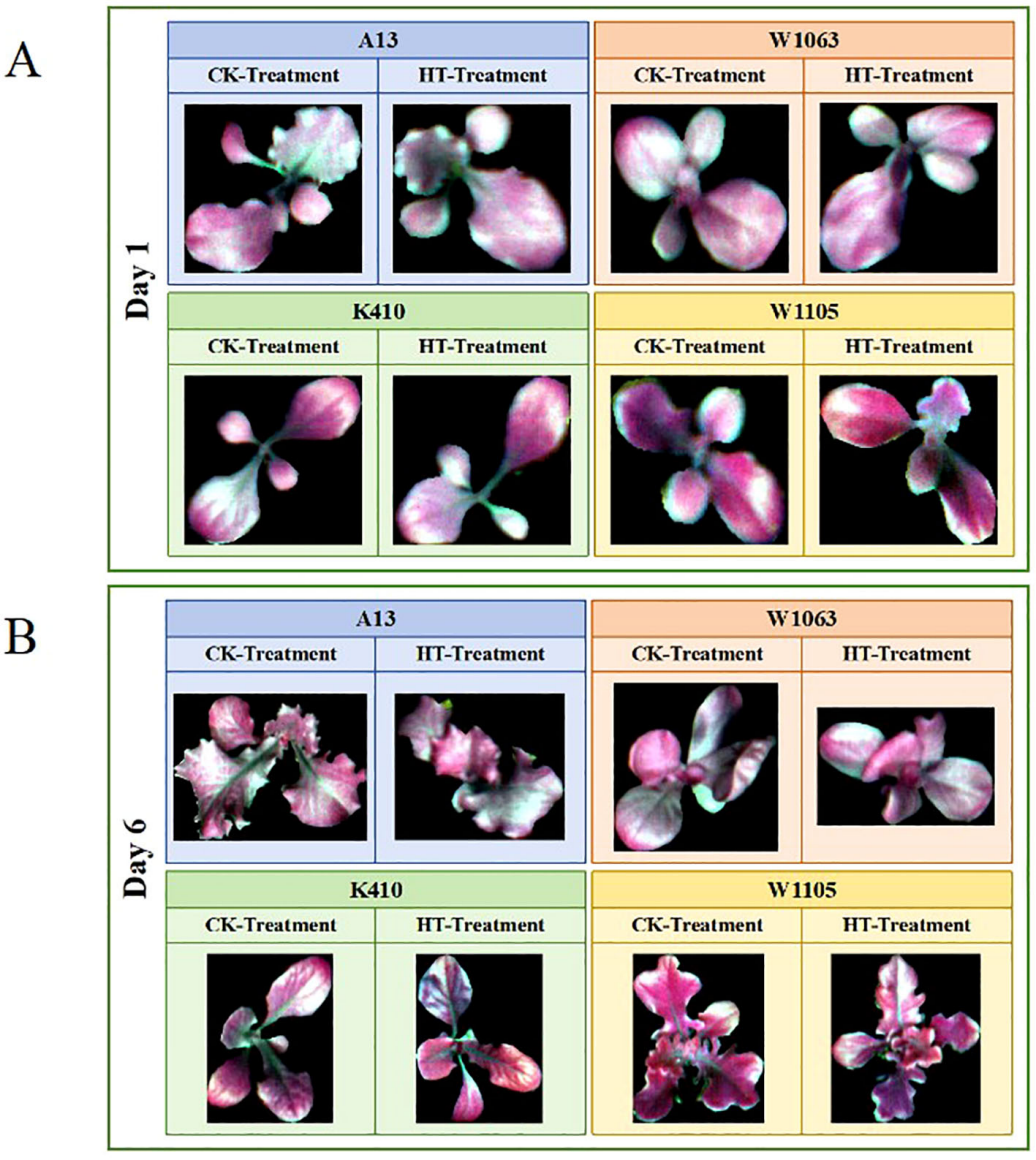
False-color hyperspectral images of four lettuce varieties on the first day (day 1) and sixth day (day 6) of cultivation under different temperature treatments: **(A)** day 1 and **(B)** day 6.

**Figure 3** shows representative false-color hyperspectral images of the four lettuce varieties under CK- and HT-treatment conditions on Day 1 and Day 6. These images visually illustrated the phenotypic status of lettuce plants at the early and later stages of temperature treatment. On Day 1, observable differences in leaf morphology, including leaf shape, leaf margin structure, and texture patterns, were mainly found among different lettuce varieties. These differences were regarded as inherent varietal morphological characteristics rather than direct responses to temperature treatment. In contrast, visible phenotypic differences between CK- and HT-treated plants within the same variety were still limited at this early stage, indicating that heat-induced symptoms had not yet become apparent. On Day 6, treatment-related visual differences became more evident after prolonged exposure to high temperature. In particular, the heat-sensitive varieties A13 and W1063 under HT-treatment showed reduced leaf expansion, paler leaf color, and varying degrees of leaf-edge curling compared with their corresponding CK-treated plants. By contrast, the heat-tolerant varieties K410 and W1105 exhibited relatively smaller visual changes under HT-treatment.

### 2.5 Dynamic selective peak transformer

**Figure 4:**
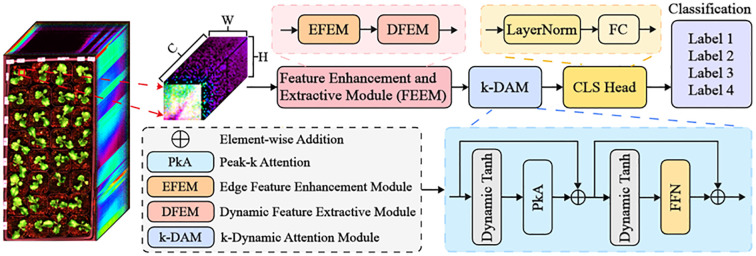
Structure of dynamic selective peak transformer (DSPformer).

To address the complexity of lettuce hyperspectral image features and minimal early-stage differences, we propose the Dynamic Selective Peak Transformer (DSPformer). As shown in **Figure 4**, the model’s core components include the Feature Enhancement and Extractive Module (FEEM) — comprising Edge Feature Enhancement Module (EFEM) and Dynamic Feature Extractive Module (DFEM) — and the k-DAM module. Finally, the extracted image features are classified through a classification head. The subsequent sections detail the architectural design of DSPformer.

#### 2.5.1 Feature enhancement and extraction module

##### 2.5.1.1 Edge feature enhancement module

**Figure 5:**
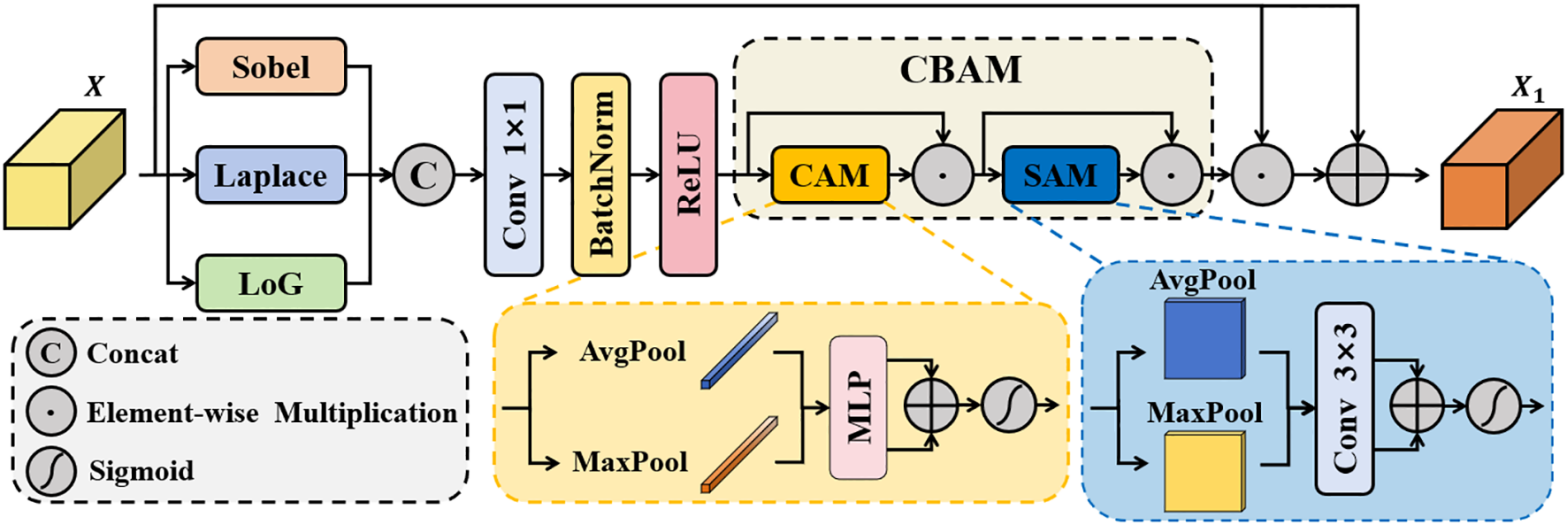
Structure of edge feature extractive attention module (EFEM).

Lettuce varieties may exhibit inherent differences in leaf edges, shapes, and texture patterns. In this study, these morphological characteristics were not regarded as direct evidence of heat-treatment responses, but as potential spatial variations that the model needs to handle when extracting heat-response-related spatial-spectral features. To improve the model’s learning of edge features, we propose the EFEM, which integrates multiple edge operators to direct the network toward edge regions and enhance perception of critical local features, with its structural design depicted in **Figure 5**.

Let $X\in {\mathbb{R}}^{C\times H\times W}$ denotes the input hyperspectral patch. The input *X* is processed through Sobel (Sobel and Feldman, 1968), Laplace (Van Vliet et al., 1989), and LoG (Marr and Hildreth, 1980) operators to extract multi-level edge features. It should be noted that LoG cannot fully replace the Laplace operator. The Laplace operator is sensitive to high-frequency information but may introduce spurious edges. LoG combines Gaussian smoothing with the Laplace operator, which suppresses noise but can blur edges and lose high-frequency details. Combining Sobel, Laplace, and LoG operators provides a richer and more stable edge representation. Along the channel dimension, outputs from the three operators are concatenated. A 1×1 convolution $Conv_{1\times 1}\left(\cdot \right)$ reduces the channels from 3*C* to *C*, ensuring dimensional consistency. Normalization and ReLU activation are applied to obtain an initial fused feature representation that integrates multiple edge information The process is formulated as Equation 1.

(1) $$X^{\prime }=ReLU\left(BN\left(Conv_{1\times 1}\left(Concat\left(Sobel\left(X\right),Laplace\left(X\right),LoG\left(X\right)\right)\right)\right)\right),$$

Hyperspectral images cover multiple bands, including visible (VIS) and near-infrared (NIR) regions. During the early stage of stress experiments, VIS bands show limited differences, whereas NIR bands can reveal subtle leaf changes earlier (Osco et al., 2019). Considering that different spectral bands respond differently to various operators in edge feature extraction, we introduce the Convolutional Block Attention Module (CBAM) (Woo et al., 2018). CBAM adaptively assigns weights to fused features through channel-wise and spatial attention modules, increasing the model’s focus on critical edge information and improving the discernible capacity and robustness of feature representations. The computation is formulated as follows (Equation 2):

(2) $$X^{\prime \prime }=\left(X^{\prime }⊙CAM\left(X'\right)\right)⊙SAM\left(X^{\prime }⊙CAM\left(X'\right)\right),$$

here, CAM(·) and SAM(·) denote the channel attention module and spatial attention module, respectively. EFEM then conducts element-wise product computation for *X* and *X*″, adaptively enhance prominent regions while suppressing irrelevant ones. The resulting product is added to *X* to form a residual connection, which preserves complete low-level feature information while introducing attention-based selectivity, ensuring a balance between feature focus and information integrity This relationship can be expressed as Equation 3:

(3) $$X_{1}=X+X⊙X^{\prime \prime },$$

where *X*_1_ represents the edge-enhanced spatial-spectral feature output by EFEM and is subsequently fed into the DFEM module.

##### 2.5.1.2 Dynamic feature extractive module

**Figure 6:**
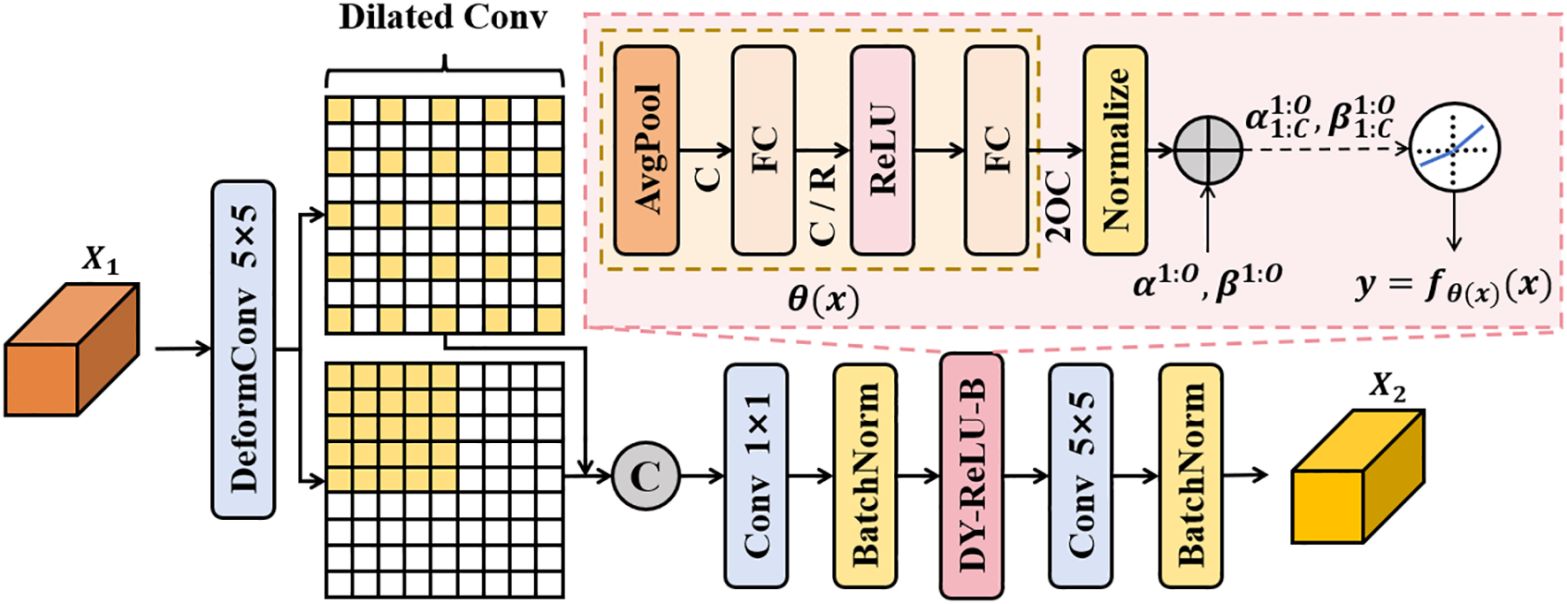
Structure of dynamic feature extractive module (DFEM).

The structure of DFEM is shown in **Figure 6**. The input $X_{1}\in {\mathbb{R}}^{C\times H\times W}$ proceeds via a 5×5 deformable convolution layer $DeformConv_{5\times 5}\left(\cdot \right)$ to dynamically extract image features. The initially extracted feature map is then fed into dilated convolution layers (Dilated Conv), where 5×5 kernels with dilation rates of 1 and 2 model correlations between different pixels, broadening the convolution range and extracting features at different scales. Across the depth dimension, outputs from the two dilated convolution routes are concatenated and fused via a 1×1 convolution layer $Conv_{1\times 1}\left(\cdot \right)$ followed by BatchNorm. This integration allows the network to simultaneously model similarities and differences between pixels, further enhancing discriminative capability as formulated in Equations 4 and 5:

(4) $$X_{1}^{\prime }=DeformConv_{5\times 5}\left(X_{1}\right),$$

(5) $$X_{1}^{fuse}=BatchNorm\left(Conv_{1\times 1}\left(\left[Conv_{5\times 5}^{r=1}\left(X_{1}^{\prime }\right);Conv_{5\times 5}^{r=2}\left(X_{1}^{\prime }\right)\right]\right)\right),$$

**Figure 7:**
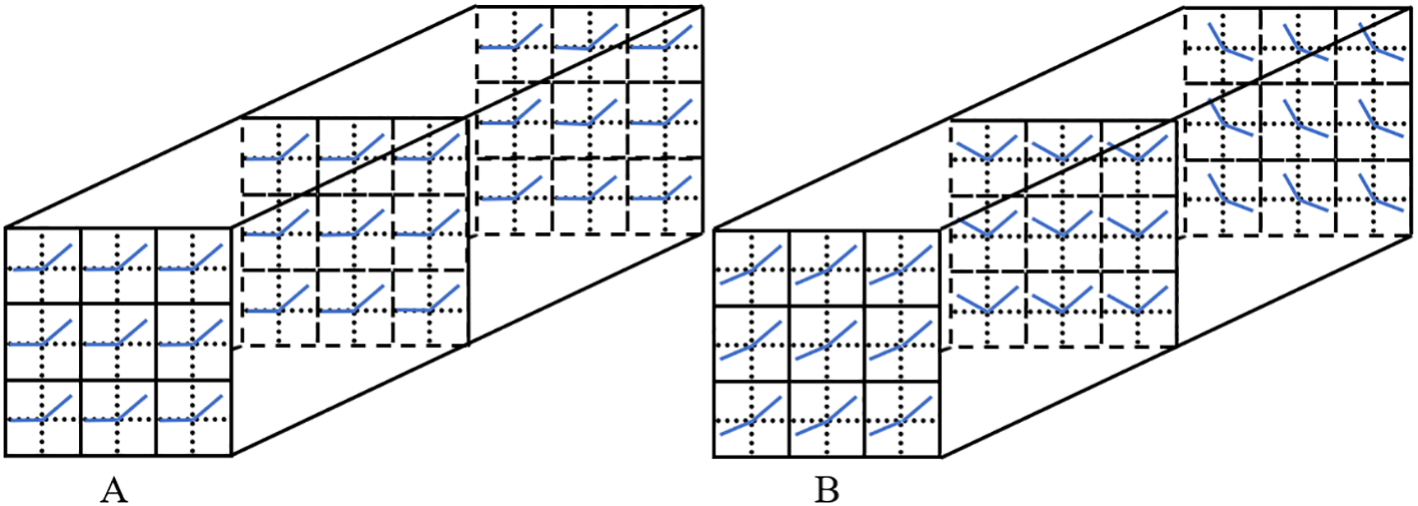
Results of two activation functions: **(A)** ReLU and **(B)** DY-ReLU-B.

HSI contains multiple channels with diverse features, and applying a uniform ReLU activation function across all channels may weaken some discriminative spectral features, as shown in **Figure 7A**. To address this issue, we introduce Dynamic ReLU (DY-ReLU-B) (Chen et al., 2020), with activation results shown in **Figure 7B**. DY-ReLU-B learns a unique activation function for each input channel, better capturing inter-channel variations and diverse spectral distributions.

To reduce the computational cost of subsequent modules, DFEM compresses the spatial size of the feature map through a 5×5 convolution layer followed by BatchNorm, while further extracting discriminative features. The resulting feature map is denoted as X_2_, which represents the output of DFEM and is subsequently fed into the k-DAM module, as shown in Equation 6:

(6) $$X_{2}=BatchNorm\left(Conv_{5\times 5}\left(DY-ReLU-B\left(X_{1}^{fuse}\right)\right)\right),$$

#### 2.5.2 k-dynamic attention module

##### 2.5.2.1 k-DAM structure

**Figure 8:**
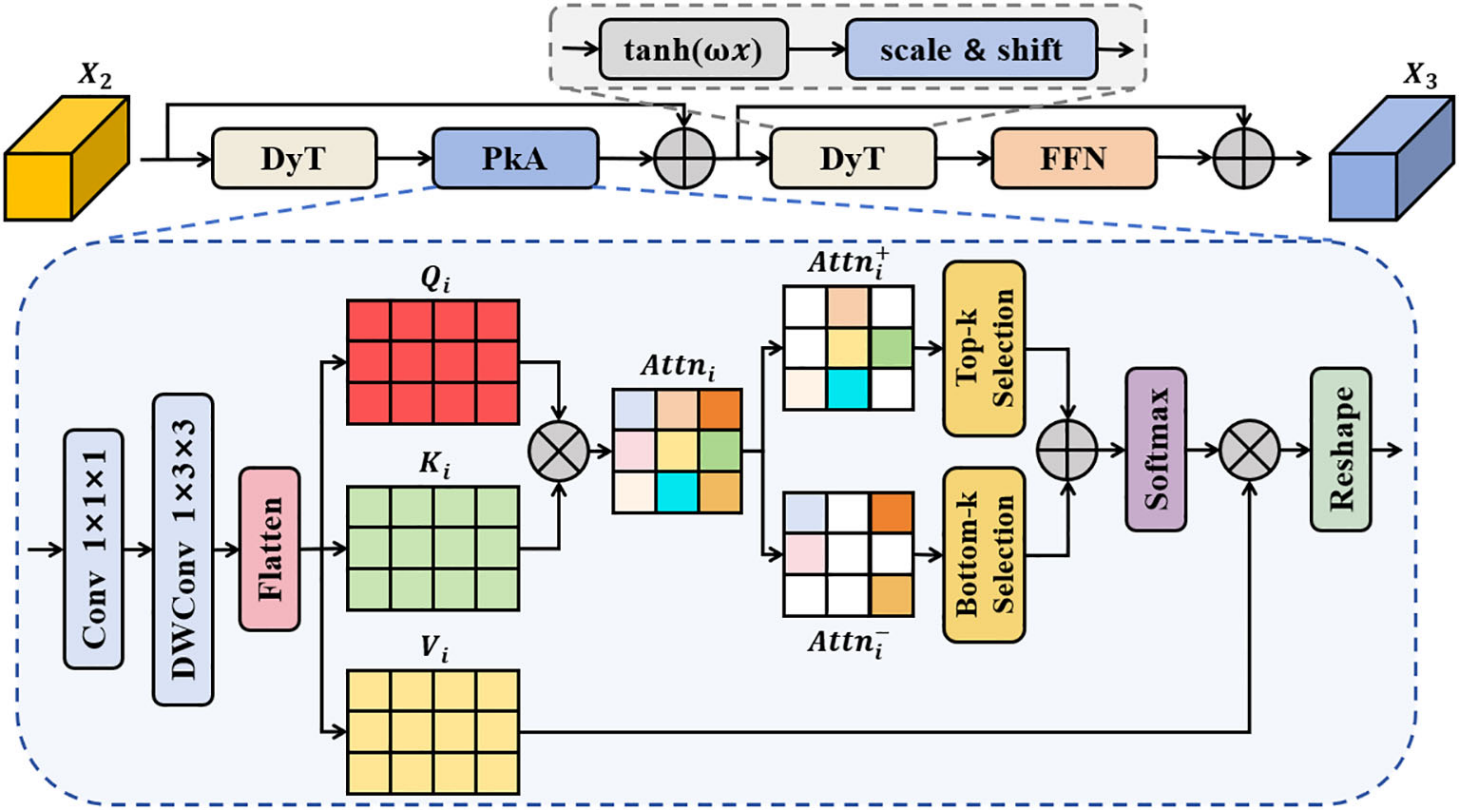
Structure of k-dynamic attention module.

The structure of k-DAM is shown in **Figure 8**. To address the instability of LayerNorm in high-dimensional data and its additional computational cost, we introduce Dynamic Tanh (DyT) (Zhu et al., 2025) as a replacement for LayerNorm. Additionally, we propose the Peak-k Attention Mechanism (PkA) as an alternative to conventional self-attention to reduce redundant information aggregation in hyperspectral feature modeling. The feature map *X*_2_, obtained from the preceding DFEM module, serves as the input to k-DAM. Within k-DAM, *X*_2_ is first processed by DyT and the proposed PkA module to capture selectively enhanced spatial-spectral dependencies. The attention-enhanced representation is then combined with the original input through a residual connection and further refined by a feed-forward network (FFN). The final output of k-DAM is denoted as *X*_3_, which is then used for subsequent classification. The encoding process of k-DAM isformulated as Equation 7:

(7) $$X_{3}=X_{2}+PkA\left(DyT\left(X_{2}\right)\right)+FFN\left(DyT\left(X_{2}+PkA\left(DyT\left(X_{2}\right)\right)\right)\right),$$

##### 2.5.2.2 Peak-k attention

In traditional self-attention mechanisms, the Softmax function converts raw attention scores into non-negative attention weights. As a result, irrelevant tokens may still receive non-zero attention weights, leading to noise accumulation (Ye et al., 2024). Specifically, in shallow Vision Transformer networks, the accumulation of small positive attention weights can shift the overall attention distribution, thereby masking key features and impairing model performance (Chen et al., 2023). In addition, before Softmax normalization, strongly negative attention scores may carry useful information about inverse or contrastive relationships between features. However, small and noisy negative scores may blur the distinction between informative negative correlations and irrelevant fluctuations, weakening the discriminative capacity of the network.

To alleviate this challenge, the proposed PkA introduces two trainable retention ratios that control the preserved proportions of positive-score and negative-score components, allowing finer selection of positive and negative attention scores in the raw attention matrix before softmax normalization. The model is thus enabled to recognize the most salient and least essential tokens within each head, ignoring other irrelevant information, reducing redundant computations, and improving overall model performance.

The network structure of PkA is shown within the blue rectangular box in **Figure 8**. First, a 3D pointwise convolution $Conv_{1\times 1\times 1}\left(\cdot \right)$ and a 3D depthwise separable convolution $DWConv_{1\times 3\times 3}\left(\cdot \right)$ are employed to encode spatial-spectral features and generate the corresponding queries, keys, and values. To facilitate subsequent self-attention computation, the model rearranges the three-dimensional tensors *Q_i_*, *K_i_* and *V_i_* into two-dimensional matrices with shape (*H* × *W*) × *C* via a flattening operation, where *i* represents the *i*-th head. *Q_i_* and *K_i_* are combined via a dot product to form the attention matrix *Attn_i_*, which is then divided into two matrices: $Attn_{i}^{+}$ and $Attn_{i}^{-}$. $Attn_{i}^{+}$ and $Attn_{i}^{-}$ contain only the positive and negative elements of *Attn_i_*, respectively. The Top-k Selection and Bottom-k Selection are applied to filter the elements to retain in each row of $Attn_{i}^{+}$ and $Attn_{i}^{-}$, as described in Equations 8 and 9:

(8) $${\left(Attn_{i}^{+}\right)}_{bs}=\{\begin{array}{c} {\left(Attn_{i}^{+}\right)}_{bs},if\;{\left(Attn_{i}^{+}\right)}_{bs}\geq (Attn_{i}^{+}{)}_{b}^{k^{+}}\\ -\infty ,otherwise \end{array},$$

(9) $${\left(Attn_{i}^{-}\right)}_{bs}=\{\begin{array}{c} {\left(Attn_{i}^{-}\right)}_{bs},\;if\;{\left(Attn_{i}^{-}\right)}_{bs}\leq (Attn_{i}^{-}{)}_{b}^{k^{-}}\\ -\infty ,otherwise \end{array},$$

Here, *b* and *s* represent the elements at the intersection of row *b* and column *s*, while *k*^+^ and *k*^−^ are two trainable retention ratios applied to the two matrices. ${\left(Attn_{i}^{+}\right)}_{b}^{k^{+}}$ denotes the top *k*^+^ % values taken from the *b*-th row of $Attn_{i}^{+}$ sorted after descending sorting, while ${\left(Attn_{i}^{-}\right)}_{b}^{k^{-}}$ refers to the bottom *k*^−^ % values extracted from the *b*-th row of $Attn_{i}^{-}$ sorted in descending order. Unlike the top-k mechanism, which uses a single fixed threshold *k*, both *k*^+^ and *k*^−^ are learnable parameters in the model.

The sum of $Attn_{i}^{+}$ and $Attn_{i}^{-}$ is denoted as $Attn_{i}^{\prime }$, which is activated using the Softmax(·) function. By multiplying with *V_i_*, the result of a single attention head is computed. The results of multipleheads are concatenated and reshaped back into a three-dimensionaltensor to produce the final feature map output, as shown in Equation 10:

(10) $$X_{PkA}=Reshape\left(Concat\left(Attn_{0}^{\prime }\times V_{0},\ldots ,Attn_{i}^{\prime }\times V_{i},\ldots ,Attn_{h}^{\prime }\times V_{h}\right)\right),$$

Here, *X_PkA_* denotes the output feature of the PkA module after multi-head attention aggregation.

#### 2.5.3 Dynamic focal loss

##### 2.5.3.1 Dynamic τ-balanced cross entropy

As shown in **Table 1** of Section 2.3, the SAGC Lettuce dataset has an imbalanced class distribution. To address this issue, we improve Focal Loss by introducing a learnable class-reweighting factor τ, which enables the network to adaptively adjust class-wise learning emphasis during training. The initial value of τ is typically set as the inverse of the class normalization frequency, assigning higher weights to underrepresented classes to balance the class distribution. The dynamic t-balanced cross-entropy is formulated as Equation 11:

**Table 1:** Number of categories in the SAGC lettuce dataset.

Index	Class	Train set	Validation set	Test set
0	CTS	4110	108	187
1	HTS	2454	64	108
2	CTT	2370	58	107
3	HTT	2142	67	101

(11) $$\tau -CE\left(p,t\right)=-\tau_{t}\log\left(p_{t}\right)=-\tau_{t}log\left(\frac{e^{z_{t}}}{\sum_{j=0}^{J-1}e^{z_{j}}}\right),$$

here, *p* signifies the normalized network’s determined score for each class, *t* denotes the target label, *J* denotes the total classes, and *z_t_* is the logit value of the *t* class. During optimization, τ is updated through backpropagation and constrained to remain positive through normalization, ensuring the validity and stability of class-wise reweighting.

##### 2.5.3.2 Dynamic γ-adjusted cross entropy

As described in Section 2.4, during the early stages of the experiment, the heat stress effects on each group were minimal, and the differences between classes were not significant, leading to potential confusion. The validation-set confusion matrix helps identify easily confused classes, providing a basis for the adaptive adjustment of the loss function. To balance learning, we introduce a confusion rate, *confuse*, to construct an adaptive *γ*, guiding the model to assign greater focus to easily confounded classes and enabling a more balanced allocation of learning emphasis. The r for each class is calculated as follows (Equation 12):

(12) $$\gamma_{t}=\gamma_{base}+\varphi \times confuse_{t}=\gamma_{base}+\varphi \times \left(\gamma_{the}-\frac{CM_{tt}}{\sum_{j=0}^{J-1}CM_{tj}}\right),$$

Where *CM_ij_* indicates the sample count for true label *t* classified into category *j* in the confusion matrix. *γ_base_* and *γ_the_* are hyperparameters, representing the base value and the confusion rate threshold, which are set to 2 and 0.95, respectively, based on empirical experience. The confusion matrix is computed on the validation set and updated once after each training epoch, thereby avoiding information leakage from the test set during optimization. *φ* serves as a learnable hyperparameter governing the impact of *confuse_t_* over *γ_t_*, allowing flexible adjustment of the model’s focus on easily confused samples. In this study, *φ* is constrained within the range of [1,2] to ensure that *γ_t_* remains positive throughout training, even when the *confuse_t_* becomes negative. Empirically, the proposed DyFocal loss exhibited stable convergence under the adopted parameter settings, indicating limited sensitivity to moderate variations of *γ_base_* and *γ_the_*. The dynamic r-Adjusted cross-entropy is defined in Equation 13:

(13) $$\gamma -CE\left(p,t\right)=-{\left(1-p_{t}\right)}^{\gamma_{t}}\log\left(p_{t}\right)=-{\left(1-p_{t}\right)}^{\gamma_{t}}\log\left(\frac{e^{z_{t}}}{\sum_{j=0}^{J-1}e^{z_{j}}}\right),$$

In the experiment, the γ−CE(p,t) with the τ-balanced variant is used as the final dynamic focal loss function, termed DyFocal, which is formulated in Equation 14:

(14) $$DyFocal\left(p_{t}\right)=-\tau_{t}{\left(1-p_{t}\right)}^{\gamma_{t}}\log\left(p_{t}\right),$$

$DyFocal\left(p_{t}\right)$ combines dynamic τ-balancing and dynamic γ-adjustment, which alleviates class imbalance while improving robustness to inter-class similarity and difficult samples during training. This improves classification stability under class imbalance and inter-class similarity in the current dataset setting.

### 2.6 Evaluation indicators

The classification performance is evaluated quantitatively via four conventional indices: Accuracy (Acc), Recall (R), Precision (P), and F1-Score (F1). Each sample is categorized as one of the following: True Positive (TP), False Positive (FP), True Negative (TN), and False Negative (FN).

Accuracy evaluates the fraction of correct predictions across the entire dataset, as defined by The process is formulated as Formula 15:

(15) $$Accuracy=\frac{TP+TN}{TP+TN+FP+FN},$$

Recall quantifies the model’s detection ratio for *TP*, as shown in The process is formulated as Formula 16.

(16) $$Recall=\frac{TP}{TP+FN},$$

Precision quantifies the model’s predictive accuracy for TP, as shown in see (Formula 17) for details

(17) $$Precision=\frac{TP}{TP+FP},$$

F1-Score is the harmonic mean of precision and recall, gauging the network’s comprehensive classification behavior, as shown in see (Formula 18) for details

(18) $$F1-Score=2\times \frac{Recall\times Precision}{Recall+Precision},$$

In addition, Parameters (Params), Floating-point Operations (FLOPs), and Train Time (TT) are employed to systematically quantify the model’s behavior and efficiency. Params measures the model’s complexity, FLOPs reflect the computational load, and TT evaluates the training time of the model.

### 2.7 Implementation details and experimental settings

All experiments were carried out on a server-based deep learning setup. The system was equipped with a 13th Gen Intel(R) Core(TM) i9-13900KF processor running at 3.00 GHz, 32 GB of RAM, and an NVIDIA GeForce RTX 4080 GPU with 16 GB of VRAM. The software stack included Windows 11, Visual Studio Code, Python 3.10, and PyTorch 2.3. The training hyperparameters for the model are summarized in **Table 2**.

**Table 2:** Model training hyperparameter settings.

Train epoch	Batch size	Optimizer	Learning rate	Weight decay	Dropout
50	16	AdamW	1*e*-4	1*e*-5	0.3

## 3 Results

### 3.1 Spectral reflectance characteristics under heat treatment

**Figure 9:**
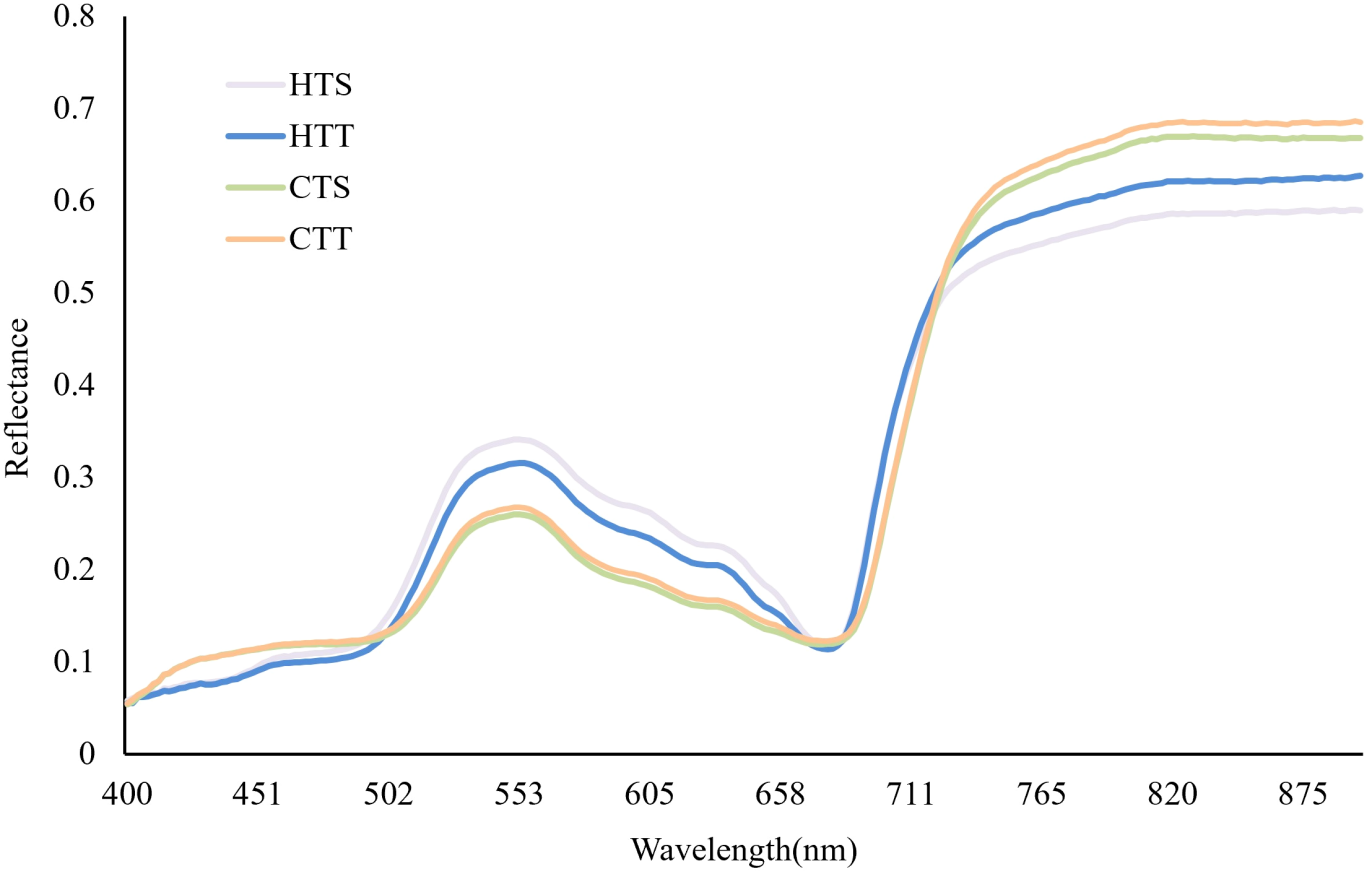
The spectral reflectance of four lettuce varieties on the sixth day under different cultivation environments.

To provide spectral evidence for early heat-response classification, the mean reflectance spectra of the four lettuce groups on Day 6 were analyzed, as shown in **Figure 9**. All groups exhibited typical vegetation spectral characteristics across the visible and near-infrared wavelength regions, including relatively low reflectance in the visible region, a reflectance peak near the green band, an absorption valley around the red region, and a rapid increase in reflectance across the red-edge region. Although the overall spectral shapes were similar among the four groups, clear differences in reflectance magnitude were observed between CK- and HT-treated plants.

In the visible region, especially from approximately 520 to 650 nm, the HT-treated groups showed higher reflectance than their corresponding CK groups. This difference was more pronounced in heat-sensitive lettuce, where HTS exhibited higher reflectance than CTS around the green to red wavelength range. In contrast, in the near-infrared region after the red-edge rise, the reflectance of HT-treated plants was clearly lower than that of CK-treated plants. The reduction was particularly evident in HTS, which showed the lowest near-infrared reflectance among the four groups, whereas CTT showed the highest reflectance in this region. These results indicate that the near-infrared region, especially around 800–880 nm, contains important information for distinguishing lettuce heat-response categories.

The observed spectral differences may be related to heat-induced changes in leaf physiological and structural properties. Reflectance changes in the visible region may be associated with pigment-related absorption characteristics, whereas the decrease in near-infrared reflectance may reflect changes in leaf internal structure, cell scattering properties, or water-related status under heat stress. Therefore, the spectral response patterns shown in **Figure 9** provide additional support for the use of hyperspectral imaging to capture heat-stress-sensitive features that are difficult to identify from visual phenotypes alone. However, because direct physiological measurements such as chlorophyll content, photosynthetic parameters, or leaf water content were not included in this study, the physiological interpretation of these wavelength regions remains indirect and should be further validated in future work.

### 3.2 Comparison of classification results with different *len* and *ϵ* values

To investigate the influence of key preprocessing parameters, namely patch length *len* and pixel coverage threshold *ϵ*, on the performance of the proposed framework, a set of preprocessing-parameter sensitivity experiments was conducted under the same training protocol. In addition to commonly used patch sizes, *len* = 73 was also evaluated because it corresponds to the minimum measured edge length among the raw lettuce images, thereby representing the largest patch size that could still be consistently applied to all samples without excessive truncation. After model training, each candidate configuration was evaluated on the same test subset described in Section 2.3, and the results are summarized in **Table 3**.

**Table 3:** Sensitivity analysis of patch length *len* and pixel coverage threshold *ϵ*.

*len*	*ϵ* (%)	Acc (%)	R (%)	P (%)	F1 (%)
16	99	71.60	72.14	70.50	71.31
32	95	73.91	73.63	71.99	72.80
64	70	93.85	92.97	94.52	93.73
64	80	**96.22**	**95.55**	**96.35**	**95.95**
73	75	95.71	95.34	96.13	95.74
73	80	89.70	89.69	88.67	89.18

The bold values identify the best patch-length/coverage-threshold setting.

**Figure 10:**
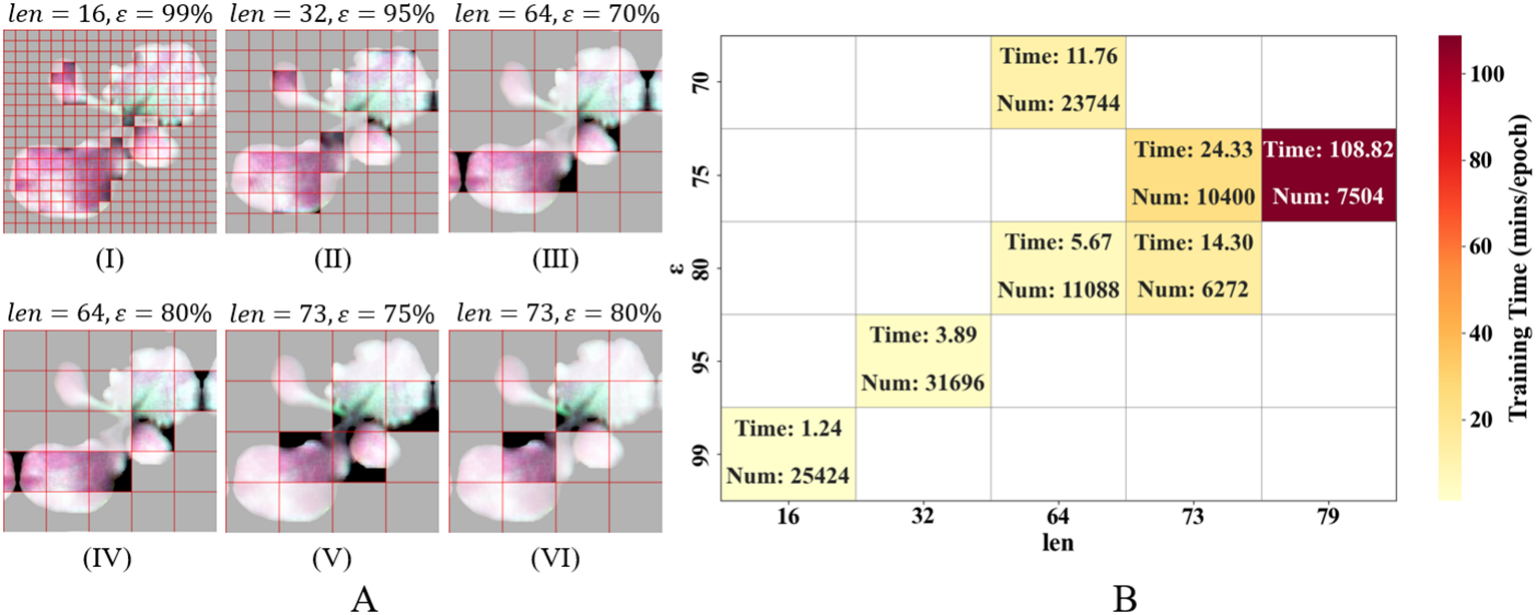
Phenotypic plots and quantitative results under different parameter combinations. **(A)** The image is segmented based on different combinations of *len* and *ϵ*, and the transparent area represents the discarded image block; **(B)** The number of training sets and model training time under different combinations of *len* and *ϵ.*.

The results in **Table 3** show that both smaller and larger values of *len* lead to varying degrees of performance degradation. When hyperspectral images are divided into smaller blocks, key detailed features, such as edges and leaf veins, are lost [see **Figure 10A(I), (II)**], resulting in the lowest classification accuracy. When *len* is large, cutting the image may directly cause feature loss [see **Figure 10A(VI)**], reducing the number of available training samples and increasing training time (see **Figure 10B**). When *len* is 64 and i is 80%, the model exhibits achieved the highest test performance under the current patch-based protocol, suggesting a favorable balance across all metrics.

### 3.3 Classification results of multiple models on SAGC lettuce dataset

To evaluate the performance of DSPformer in the early classification of lettuce samples with different heat-tolerance labels under controlled treatments, this study compares it with several recent advanced classification models, including 2D-CNN (Lee and Kwon, 2016), 3D-CNN (Hamida et al., 2018), SSFTT (Sun et al., 2022), CAEVT (Zhang et al., 2022), MorphFormer (Roy et al., 2023), GSCViT (Zhao et al., 2024), TNCCA (Wang et al., 2024), and DSFormer (Xu et al., 2025).

**Table 4** lists the experimental results, highlighting the best metric values in bold. The proposed DSPformer enhances edge and texture feature learning through FEEM, adaptively learns effective pixels, and integrates multi-scale receptive fields. By aggregating global information and selecting key features, k-DAM contributes to improved classification performance under the current experimental setting. The Acc, R, P, and F1 scores of DSPformer are 96.22%, 95.55%, 96.35%, and 95.95%, respectively, showing improvements of 1.50%, 0.92%, 2.37%, and 1.64% compared to the second-best model, DSFormer. **Table 4** compares different models in terms of Params, GFLOPs, and TT. DSPformer has 7.19M parameters and 193.15G GFLOPs, which are higher than most of the comparison models. However, its training time is only 5.67 minutes, which is close to that of several lightweight models and even shorter in some cases. These results indicate that DSPformer achieves strong representational capacity with acceptable training time, although its parameter size and GFLOPs remain higher than those of several lightweight models.

**Table 4:** Performance of various classification approaches.

Model	Acc (%)	R (%)	P (%)	F1 (%)	Params (M)	GFLOPs (G)	TT (min)
2D-CNN	79.58	75.27	76.79	76.02	53.11	50.28	3.72
3D-CNN	84.60	83.13	83.75	83.44	0.30	54.69	3.96
SSFTT	86.46	81.82	90.59	85.99	1.10	65.97	3.81
CAEVT	87.40	86.96	86.51	86.73	3.63	1124.71	10.71
MorphFormer	91.11	92.06	90.17	91.11	0.29	26.57	3.89
GSCViT	88.40	86.66	86.34	86.50	0.13	8.26	3.73
TNCCA	89.20	87.15	87.52	87.34	0.98	1.27	4.31
DSFormer	94.80	94.68	94.12	94.40	1.03	21.46	6.58
DSPformer	**96.22**	**95.55**	**96.35**	**95.95**	7.19	193.15	5.67

The bold values represent the best model performance.

**Figure 11:**
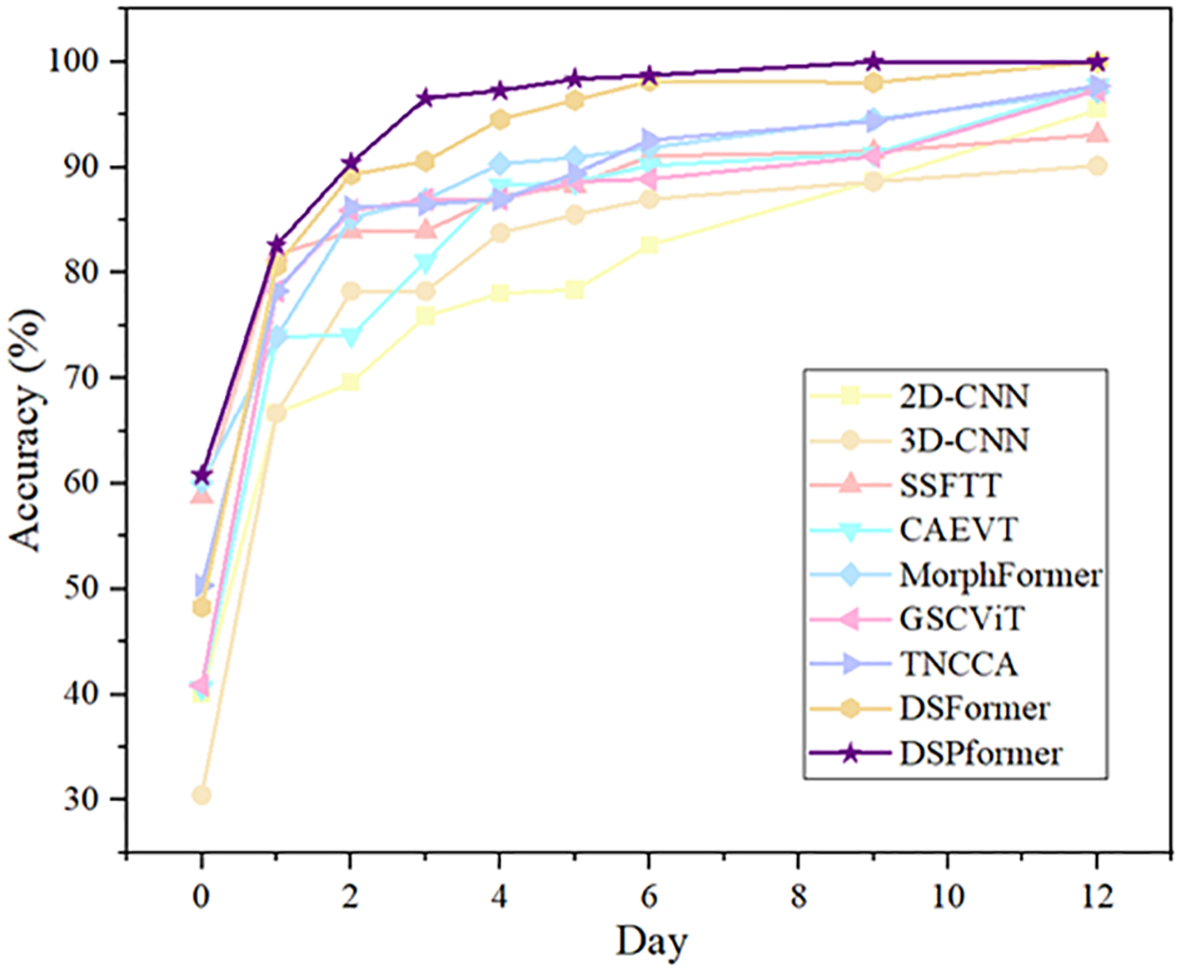
Classification accuracy of different models on daily datasets.

In addition to overall classification accuracy, the temporal separability of lettuce samples with different heat-response categories is also important for evaluating the proposed framework. **Figure 11** presents the classification accuracy of different models at each acquisition time point, where Day 0 represents the pre-treatment baseline before high-temperature stress was imposed. At Day 0, DSPformer achieved an accuracy of 60.76%, only slightly higher than the second-best MorphFormer (60.12%). Since heat-treatment-induced responses were not expected at this stage, the limited classification accuracy indicates that the four categories could not be reliably distinguished based solely on inherent varietal morphology, such as leaf shape, leaf margin structure, or texture patterns. After the high-temperature treatment began, the classification performance of DSPformer increased markedly, reaching 82.61% on Day 1 and 96.55% on Day 3, whereas the best-performing comparison model achieved only 90.59% on Day 3. This rapid improvement suggests that treatment-related hyperspectral and spatial-spectral differences became progressively more detectable during the early stage of heat exposure. Notably, this improvement occurred before the visible heat-stress phenotypes became apparent on Day 6, indicating that DSPformer could capture subtle heat-response-related cues that were difficult to identify from visual morphology alone. These results further support that the proposed framework did not simply rely on pre-existing morphological differences among varieties, but learned discriminative spatial-spectral features associated with the developing heat response under the current controlled experimental setting.

### 3.4 Robustness and statistical significance analysis under plant-level partition

To further evaluate the robustness and reproducibility of DSPformer under a more rigorous evaluation protocol, additional experiments were conducted using a plant-level partition strategy. Unlike conventional patch-level random splitting, all patches extracted from the same lettuce plant were assigned exclusively to a single subset, thereby preventing correlated samples from appearing simultaneously in training and testing sets. Five independent experiments were performed using random seeds 2022-2026. The mean and standard deviation of the evaluation metrics are summarized in **Table 5**. Compared with the original patch-level evaluation, the overall performance of all models decreased, indicating that the plant-level protocol provides a more challenging and realistic assessment of model generalization.

**Table 5:** Statistical robustness under plant-level partition.

Model	Acc (%)	R (%)	P (%)	F1 (%)
DSFormer	92.34 ± 0.25	92.94 ± 0.26	92.40 ± 0.44	92.67 ± 0.31
DSPformer	93.76 ± 0.49	93.75 ± 0.54	93.26 ± 0.43	93.50 ± 0.42
p-value	<0.001	0.0054	0.0276	0.0060

Nevertheless, DSPformer consistently achieved the best performance across all evaluation metrics, obtaining an accuracy of 93.76 ± 0.49%, recall of 93.75 ± 0.54%, precision of 93.26 ± 0.43%, and F1-score of 93.50 ± 0.42%. In comparison, the strongest baseline DSFormer achieved 92.34 ± 0.25%, 92.94 ± 0.26%, 92.40 ± 0.44%, and 92.67 ± 0.31%, respectively.

To assess statistical significance, a two-tailed Welch’s t-test was conducted between DSPformer and DSFormer across the five runs. The resulting p-values were<0.001, 0.0054, 0.0276, and 0.0060 for Acc, Recall, Precision, and F1-score, respectively, indicating statistically significant improvements (p< 0.05) across all evaluation metrics. These results demonstrate that DSPformer maintains superior performance under a more stringent plant-level partition protocol while exhibiting favorable robustness and reproducibility.

### 3.5 Ablation experiment

To assess the contribution of EFEM, DFEM, k-DAM, and DyFocal, we carried out a set of ablation experiments, with the results summarized in **Table 6**. The baseline adopts a network structure with three consecutively stacked 5×5 convolutional layers to match the depth of the DSPformer model.

**Table 6:** Results of ablation experiments with different module combinations.

EFEM	DFEM	k-DAM	Loss function	Acc (%)	R (%)	P (%)	F1 (%)
✗	✗	✗	DyFocal	87.42	86.28	86.17	86.22
✓	✗	✗	DyFocal	89.12	88.24	88.76	88.5
✗	✓	✗	DyFocal	91.75	91.51	90.19	90.85
✗	✗	✓	DyFocal	90.98	90.79	90.82	90.8
✗	✓	✓	DyFocal	94.75	94.23	94.78	94.5
✓	✗	✓	DyFocal	93.77	93.08	93.74	93.41
✓	✓	✗	DyFocal	93.87	93.36	93.61	93.48
✓	✓	✓	Focal Loss	95.43	95.25	95.45	95.31
✓	✓	✓	AdaFocal	96.02	95.44	96.06	95.73
✓	✓	✓	DyFocal	**96.22**	**95.55**	**96.35**	**95.95**

The bold values represent the best ablation configuration.

Without introducing any modules, the baseline model achieves an accuracy of only 87.42%. After introducing the individual EFEM, DFEM, and k-DAM modules, the accuracy increased to 89.12%, 91.75%, and 90.98%, respectively. The experimental results demonstrate that the three design modules markedly improve the model’s classification effectiveness. Specifically, EFEM extracts subtle edge and texture features from different bands using various edge operators, and dynamically assigns weights to each band via CBAM, enabling early detection of subtle changes in lettuce. DFEM, as the main feature extraction module, integrates DeformConv and multi-scale convolutions to expand the receptive field, and uses DY-ReLU-B to learn the spectral response of each channel, capturing more valuable detail features. k-DAM focuses on relevant features and mitigates noise from less informative signals by calculating the spatial and spectral context of the most and least relevant features, thereby improving classification performance.

Further analysis of the module combination strategy shows that using any two modules together consistently improves model performance under the current setting, indicating good complementarity in feature extraction among the three modules. When DFEM and k-DAM are used together, the accuracy reaches 94.75%, indicating that the fusion of local details and global context improves the feature representation ability. When all three modules are introduced together, the model performs best across all metrics, achieving the best performance among the tested combinations, supporting the complementary roles of the proposed modules in multi-dimensional feature capture and integration. Additionally, **Table 6** reports DSPformer’s classification results using different loss functions when all three modules are employed. When using DyFocal, the model consistently ranks first across the four metrics, with AdaFocal’s performance being similar, supporting the effectiveness of DyFocal in this classification setting.

**Figure 12:**
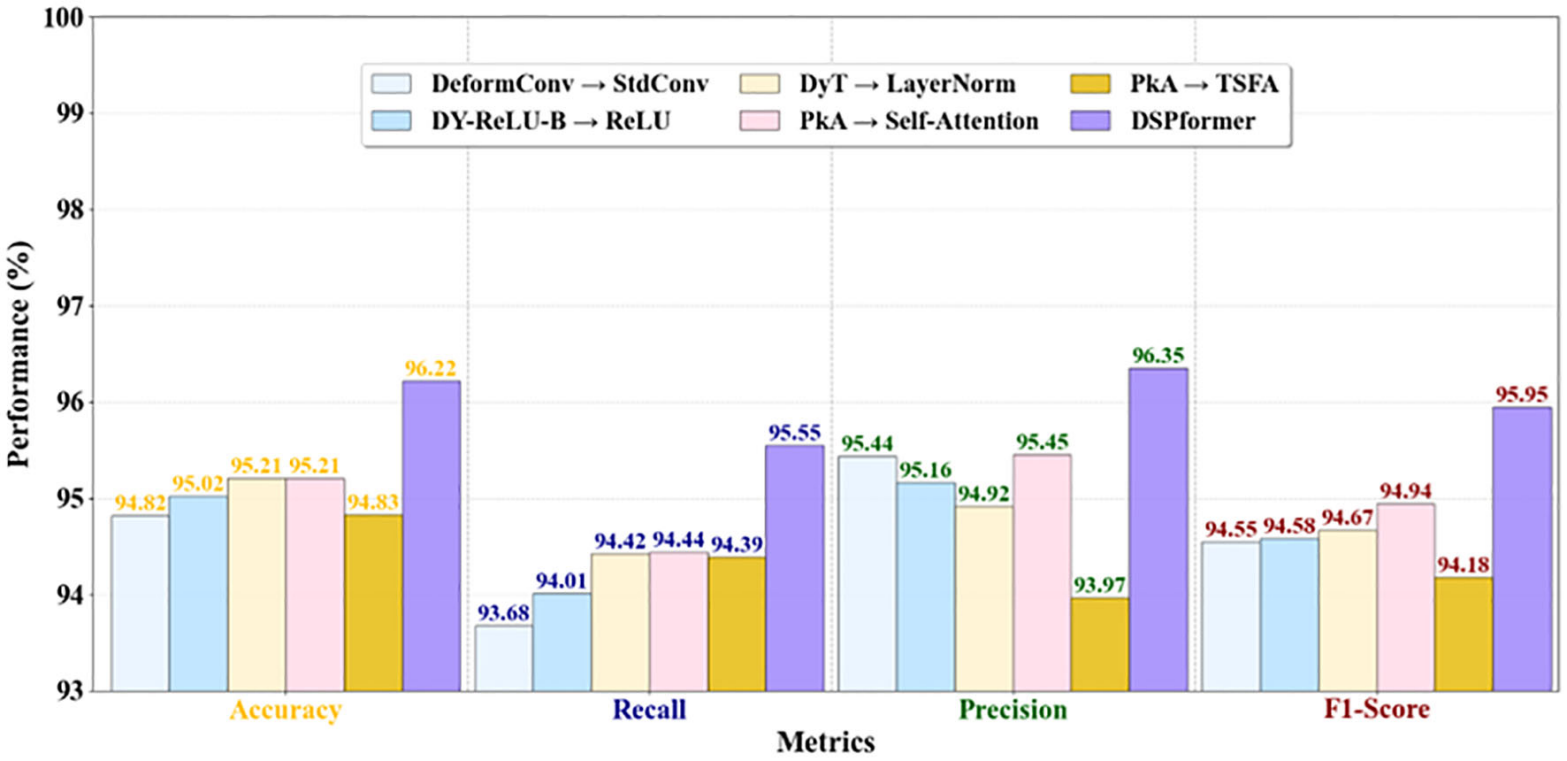
Comparison of model performance after replacing different components.

To comprehensively validate the rationale behind the module design, DeformConv in DFEM was replaced with StdConv, DY-ReLU-B with ReLU, DyT in k-DAM with LayerNorm, PkA with Self-attention, and Token Selective Fusion Attention (TSFA) based on the top-*k* mechanism (Xu et al., 2025). **Figure 12** illustrates the experimental results.

**Figure 12** visually shows the performance differences after replacing various components. The experimental results indicate that replacing the key structures of DSPformer with conventional components leads to varying degrees of performance degradation, supporting the effectiveness of each module. Among all ablation combinations, replacing PkA with Self-attention achieved the highest overall performance, while replacing it with TSFA resulted in the lowest performance. The results reflect that TSFA failed to effectively select key markers, and Self-attention, due to its global association mechanism, tends to introduce redundant information. Both perform less reliably and stably than PkA’s dual-threshold strategy. These results suggest that PkA provides a more effective sparse attention pattern than the compared alternatives.

## 4 Discussion

### 4.1 Comparative performance and reliability

The results presented in **Table 4** demonstrate that DSPformer consistently outperformed all compared CNN-based and Transformer-based methods on the SAGC Lettuce dataset. In particular, DSPformer achieved an Acc of 96.22%, exceeding the strongest baseline DSFormer by 1.42 percentage points while simultaneously obtaining the highest Recall, Precision, and F1-score. The consistent improvement across all evaluation metrics suggests that the proposed framework not only improves overall classification accuracy but also enhances the balance between minority-class recognition and prediction reliability.

The observed performance gains are consistent with findings reported in previous hyperspectral image classification studies. Existing research has shown that effective integration of local spatial structures and long-range spectral-spatial dependencies is critical for improving classification performance. CNN-based methods such as 2D-CNN and 3D-CNN are effective in extracting local features but often exhibit limited capability in modeling global contextual relationships. In contrast, Transformer-based approaches, including SSFTT, MorphFormer, GSCViT, TNCCA, and DSFormer, enhance representation learning through attention mechanisms and global feature interaction. The superior performance of DSPformer further supports this general trend and suggests that combining complementary local and global feature extraction strategies remains an effective direction for hyperspectral image analysis.

Compared with existing Transformer-based methods, the novelty of DSPformer lies not in simply increasing model complexity but in the coordinated integration of three complementary components. EFEM enhances structurally informative spectral-spatial patterns before feature learning, DFEM improves adaptive multi-scale representation capability, and k-DAM selectively suppresses redundant attention responses during feature aggregation. The resulting performance improvements were consistently observed across classification metrics and public benchmark evaluation, indicating that the proposed design effectively addresses the challenges of subtle inter-class differences and high spectral redundancy that commonly occur in hyperspectral phenotype recognition tasks.

From an application perspective, the most notable advantage of DSPformer is its ability to capture heat-response-related spatial-spectral cues at an early stage. As shown in **Figure 11**, Day 0 represents the pre-treatment baseline before high-temperature stress was imposed. At this stage, DSPformer achieved an accuracy of 60.76%, only slightly higher than the second-best MorphFormer (60.12%), indicating that the four categories could not be reliably distinguished based solely on inherent varietal morphology, such as leaf shape, leaf margin structure, or texture patterns. After high-temperature treatment began, the accuracy of DSPformer increased markedly to 82.61% on Day 1 and 96.55% on Day 3, whereas the best-performing comparison model achieved only 90.59% on Day 3. This rapid improvement, which occurred before visible heat-stress phenotypes became apparent on Day 6, suggests that DSPformer captured progressively emerging heat-response-related hyperspectral and spatial-spectral cues rather than simply relying on pre-existing morphological differences among varieties. Together with the plant-level partition results, these findings further support the robustness of the learned representations under the interference of varietal morphological variation.

Additional validation under a plant-level partition protocol (**Table 5**) further supports the reliability and robustness of the proposed framework. Although all models exhibited lower performance compared with the original patch-level evaluation, indicating a more challenging assessment scenario, DSPformer remained the best-performing method, achieving 93.76 ± 0.49% accuracy and maintaining statistically significant improvements over the strongest baseline DSFormer across all evaluation metrics (p< 0.05). These results demonstrate that the performance advantage of DSPformer cannot be solely attributed to correlations among neighboring patches, but rather reflects its ability to learn discriminative spatial-spectral representations that generalize across different lettuce plants.

### 4.2 Performance analysis under class imbalance and inter-class confusion

**Figure 13:**
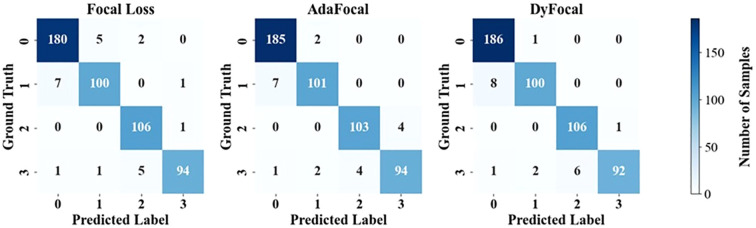
Test set confusion matrix under three loss functions.

To validate DSPformer’s ability in mitigating class imbalance and confusion within SAGC Lettuce dataset, confusion matrices were constructed for the predictions of three loss functions on the test set, as depicted in **Figure 13**.

The results indicate that DyFocal achieved favorable performance in overall classification accuracy, maintaining the highest correct recognition in Class 1 and Class 3. Under class imbalance conditions, DyFocal’s recognition accuracy for minority samples in Class 2 is similar to that of Focal Loss, but with fewer misclassifications, suggesting its advantage in balancing precision and stability. For the hard-to-classify samples in Class 4, DyFocal’s recognition rate is comparable to AdaFocal, with misclassifications mainly occurring between adjacent classes, indicating that this method effectively mitigates class confusion.

Overall, the combination of a learnable τ and a confusion rate-based γ enables DyFocal to better cope with class imbalance and difficult samples, contributing to improved classification stability under the current dataset setting.

### 4.3 Experimental results of different models on public datasets

To further examine the applicability of DSPformer on a standard hyperspectral image classification benchmark, experimental evaluation was also conducted on the publicly available Indian Pines dataset. The Airborne Visible Infrared Imaging Spectrometer (AVIRIS) collected this dataset in Indiana in 1992. It has a size of 145×145 pixels and a spatial resolution of 20m. After excluding 20 water absorption bands, 200 spectral bands covering the 400–2500 nm wavelength range were retained. Indian Pines covers 16 crop types, including corn, soybeans, and wheat.

In the experiment, 50 instances per crop class constituted the training set, except for Classes 1, 7, and 9, which contained only 15 instances each. The remaining data served as the testing set. The images were divided into 10×10-pixel blocks as input for the model. Overall accuracy (OA), average accuracy (AA), and kappa coefficient (κ) were employed to assess classification performance. OA represents the proportion of pixels that have been correctly labeled out of the total pixel count. AA indicates the average accuracy computed over every class, reflecting classification stability. κ quantifies the consistency between predictions and true labels.

**Table 7** displays the experimental results. DSPformer achieved OA, AA, and κ values of 95.21%, 97.67%, and 94.51%, respectively. Its OA and κ were slightly higher than those of the compared models, while its AA was second only to DSFormer. For several challenging categories, such as Classes 2, 10, and 11, DSPformer also achieved competitive classification accuracy.

**Table 7:** Classification performances of Indian pines dataset.

Class	2D-CNN	3D-CNN	SSFTT	CAEVT	MorphFormer	GSCVit	TNCCA	DSFormer	DSPformer
1	93.87	93.87	96.77	**100.00**	97.10	99.03	99.35	99.35	**100.00**
2	64.95	60.45	84.45	89.86	88.15	89.47	86.79	93.22	**93.98**
3	86.51	70.64	70.05	87.82	94.05	**96.53**	95.97	96.08	91.15
4	98.13	95.72	97.65	77.90	99.89	99.89	99.36	99.79	**100.00**
5	94.27	89.49	95.38	**99.29**	96.95	97.23	97.69	98.48	98.61
6	97.26	96.85	96.82	94.97	98.63	99.43	99.19	**99.68**	98.97
7	**100.00**	96.92	**100.00**	**100.00**	**100.00**	**100.00**	**100.00**	**100.00**	**100.00**
8	98.41	98.06	99.58	90.66	99.74	99.93	**100.00**	**100.00**	**100.00**
9	**100.00**	**100.00**	**100.00**	87.50	**100.00**	**100.00**	**100.00**	**100.00**	**100.00**
10	83.66	76.80	88.37	86.98	91.34	94.03	92.98	94.03	**94.79**
11	72.83	66.88	60.80	90.02	87.42	89.50	88.30	90.25	**91.60**
12	90.50	82.71	91.14	84.65	95.49	95.82	96.30	96.24	**96.50**
13	99.48	99.87	99.94	88.29	99.94	99.94	99.81	**100.00**	**100.00**
14	94.93	92.24	96.29	95.27	97.42	97.77	98.21	**98.52**	98.35
15	96.46	94.85	97.11	95.22	98.72	99.38	99.70	**99.88**	98.81
16	**100.00**	**100.00**	99.77	**100.00**	99.77	**100.00**	**100.00**	**100.00**	**100.00**
OA (%)	83.53	78.28	83.47	90.71	92.91	94.26	93.51	95.17	**95.21**
AA (%)	91.95	88.46	92.70	78.61	96.54	97.37	97.10	**97.85**	97.67
κ (%)	81.23	75.30	81.43	89.37	91.89	93.43	92.58	94.47	**94.51**

The bold values represent the best class-wise accuracy.

The successful performance of DSPformer on both the SAGC Lettuce dataset and the Indian Pines benchmark suggests that the proposed architecture has a certain degree of transferability across different hyperspectral classification scenarios. These two datasets differ substantially in spatial resolution, scene composition, and feature scale. Therefore, the results on Indian Pines should not be interpreted as direct evidence for the effectiveness of leaf-edge or micro-texture extraction mechanisms. Instead, they mainly demonstrate the applicability of the overall DSPformer framework to general hyperspectral image classification tasks.

### 4.4 Limitation analysis

First, the primary benchmark experiments in this study were conducted under a patch-level partition protocol, which facilitates direct comparison with existing hyperspectral image classification methods. To address the potential concern that correlated patches from the same lettuce plant may appear in different subsets, an additional plant-level partition experiment was performed in the revised study. Although DSPformer maintained statistically significant performance advantages under this more rigorous setting, the current evaluation was still conducted using data collected from a single experimental population under controlled environmental conditions. Therefore, further validation on independent populations, cultivation environments, growing seasons, and breeding materials is required to comprehensively assess the practical deployment capability of DSPformer.

Second, the current comparative experiments were conducted under a fixed experimental configuration. Although DSPformer demonstrated improved performance under this setting, repeated runs with multiple random seeds will be considered in future studies to further quantify performance variability and reproducibility.

Third, repeated experiments with five independent random seeds were conducted to evaluate the reproducibility and robustness of DSPformer. As shown in **Table 5**, statistical significance analysis further confirmed that the proposed method consistently outperformed the strongest baseline under the plant-level partition protocol. Nevertheless, the current statistical evaluation was based on a limited number of repeated runs. Future work will incorporate more extensive repeated experiments and additional benchmark datasets to further characterize performance variability and robustness. Third, although the proposed k-DAM module improves the selectivity of attention computation by reducing redundant responses, its sparsity strategy may still require further optimization for large-scale datasets. More efficient sparse attention mechanisms and adaptive token selection strategies will be explored to reduce computational complexity and memory consumption while maintaining discriminative capability.

Finally, DSPformer still has a relatively large parameter size compared with lightweight architectures such as GSCViT and MorphFormer. This may limit its direct deployment on resource-constrained edge devices. Therefore, future work will investigate lightweight optimization strategies, including model pruning, parameter compression, and efficient architectural redesign, to improve inference efficiency and deployment capability for practical heat-tolerant lettuce breeding applications.

## 5 Conclusion

This study demonstrates the feasibility of using hyperspectral imaging and deep learning for early-stage classification of lettuce heat responses under controlled experimental conditions. The added spectral reflectance analysis indicates that heat treatment caused detectable changes in both visible and near-infrared regions. In particular, the higher reflectance observed in the visible range and the reduced near-infrared reflectance of heat-treated plants, especially around 800–880 nm, suggest that hyperspectral imaging can capture heat-stress-related information before obvious visual symptoms are fully expressed. These spectral differences provide additional support for the biological relevance of early heat-response classification using hyperspectral data.

The proposed DSPformer achieved the best overall performance among the compared hyperspectral image classification models. Its advantage is mainly attributed to the coordinated use of edge-enhanced feature extraction, dynamic multi-scale spatial-spectral representation, selective attention-based redundancy suppression, and the DyFocal loss for handling class imbalance and inter-class confusion. The day-wise classification results further showed that DSPformer substantially improved classification accuracy after the onset of high-temperature treatment and reached high accuracy before visible heat-stress symptoms appeared on Day 6. This finding suggests that the model learned progressively emerging heat-response-related spatial-spectral cues rather than relying only on inherent varietal morphological differences.

From a practical perspective, the proposed framework provides a non-destructive and efficient approach for early screening of lettuce materials with contrasting heat-response characteristics. Such a method could help reduce the dependence on delayed manual phenotyping and provide technical support for high-throughput phenotyping-assisted heat-tolerance evaluation. The additional plant-level partition experiment further confirmed the robustness of DSPformer under a more stringent evaluation protocol, while the Indian Pines experiment suggested that the overall architecture has potential applicability to broader hyperspectral image classification tasks.

Nevertheless, several limitations remain. The current lettuce dataset was collected from a controlled experimental environment and a limited set of varieties, and direct physiological measurements such as chlorophyll content, photosynthetic parameters, and leaf water status were not included. Therefore, the physiological interpretation of key wavelength regions remains indirect. In addition, DSPformer still has higher computational complexity than some lightweight models, which may restrict its direct deployment on resource-constrained devices. Future research will focus on validating the framework across independent seasons, cultivation environments, larger breeding populations, and different stress conditions. Further integration of physiological measurements, key-band selection, model lightweighting, and field-scale phenotyping platforms will be important for promoting practical application in lettuce heat-tolerance screening and breeding.

## Data Availability

The raw data supporting the conclusions of this article will be made available by the authors, without undue reservation.
